# Recurrent Home Flooding in Detroit, MI 2012–2020: Results of a Household Survey

**DOI:** 10.3390/ijerph18147659

**Published:** 2021-07-19

**Authors:** Peter S. Larson, Carina Gronlund, Lyke Thompson, Natalie Sampson, Ramona Washington, Jamie Steis Thorsby, Natalie Lyon, Carol Miller

**Affiliations:** 1 Social Environment and Health Program, Survey Research Center, Institute for Social Research, The University of Michigan, Ann Arbor, MI 48109, USA; gronlund@umich.edu; 2 Department of Epidemiology, School of Public Health, University of Michigan, Ann Arbor, MI 48109, USA; 3 Center for Urban Studies, Wayne State University, Detroit, MI 48202, USA; Lyke@wayne.edu (L.T.); as0293@wayne.edu (R.W.); 4 Department of Health and Human Services, University of Michigan-Dearborn, 19000 Hubbard Drive, Fairlane Center South, Dearborn, MI 48126, USA; nsampson@umich.edu; 5 Healthy Urban Waters, Wayne State University, Detroit, MI 48202, USA; jamie.steis@wayne.edu (J.S.T.); natalie.lyon@wayne.edu (N.L.); ab1421@wayne.edu (C.M.)

**Keywords:** flooding, Detroit, environmental justice, climate change

## Abstract

Household flooding has wide ranging social, economic and public health impacts particularly for people in resource poor communities. The determinants and public health outcomes of recurrent home flooding in urban contexts, however, are not well understood. A household survey was used to assess neighborhood and household level determinants of recurrent home flooding in Detroit, MI. Survey activities were conducted from 2012 to 2020. Researchers collected information on past flooding, housing conditions and public health outcomes. Using the locations of homes, a “hot spot” analysis of flooding was performed to find areas of high and low risk. Survey data were linked to environmental and neighborhood data and associations were tested using regression methods. 4803 households participated in the survey. Flooding information was available for 3842 homes. Among these, 2085 (54.26%) reported experiencing pluvial flooding. Rental occupied units were more likely to report flooding than owner occupied homes (Odd ratio (OR) 1.72 [95% Confidence interval (CI) 1.49, 1.98]). Housing conditions such as poor roof quality and cracks in basement walls influenced home flooding risk. Homes located in census tracts with increased percentages of owner occupied units (vs. rentals) had a lower odds of flooding (OR 0.92 [95% (CI) 0.86, 0.98]). Household factors were found the be more predictive of flooding than neighborhood factors in both univariate and multivariate analyses. Flooding and housing conditions associated with home flooding were associated with asthma cases. Recurrent home flooding is far more prevalent than previously thought. Programs that support recovery and which focus on home improvement to prevent flooding, particularly by landlords, might benefit the public health. These results draw awareness and urgency to problems of urban flooding and public health in other areas of the country confronting the compounding challenges of aging infrastructure, disinvestment and climate change.

## 1. Introduction

Pluvial flooding and sewer overflows comprise a multifactorial public health problem that raises risks for mortality and morbidity of a host of serious health conditions. Health impacts from flooding can be physical or psychological. Physical outcomes include gastrointestinal illnesses, respiratory illnesses, skin rashes and infections and poisoning from exposure to chemical irritants [1,2,3,4,5,6,7]. Psychological distress and trauma are common among people who have experienced sudden flooding as a result of extreme weather events [8,9,10].

Flooding impacts can be felt during or after the flooding event [11]. Women, the elderly and small children are more likely to experience physical and psychological outcomes during floods whereas men are at high risk for mortality during and after flooding events [12,13,14,15,16]. Intensity of flooding has a graded impact on physical and psychological health; long term psychological effects such as anxiety, depression and PTSD can occur for several years following a flooding event [8,17,18,19]. Flooding can cause serious disruptions to the provisions of mental health treatment exacerbating existing mental health problems in people who have them [20]. Long term effects include trauma and stress-related problems as a result of displacement, economic insecurity and future housing uncertainty [21]. Mortality has been found to increase in the months following major flood events [22] and flooding impacts can be felt even by persons outside flooded areas through groundwater contamination [23].

Home flooding is a serious public health problem that has long lasting health impacts for people who experience it [24,25,26,27,28,29,30]. Microbial toxins such as molds and fungus are known to worsen respiratory problems and are associated with the development of asthma and allergic rhinitis [31,32]. Evidence from New Orleans, LA following Hurricane Katrina suggests that homes with experienced flooding were more likely to develop molds and fungus [33]. Home dampness has been found to increase the severity of symptoms for a number of respiratory conditions, including asthma and chronic bronchitis and to increase exposure to numerous types of microbial agents [34,35,36]

Climate and rainfall patterns in the Midwest region of the United States have been altered over the past five decades due to climate change [37,38]. Globally, urban land exposed to climate and flooding hazard risks is expected to increase by more than 25 percent compared with current levels [39]. Natural hazards and extreme weather events have become more common and increasingly costly in the US over the past two decades, the costliest of which have impacted urban areas such as Detroit, MI [40,41,42]. Changes in frequency and intensity of rainfall patterns can overwhelm aging infrastructure in challenged cities like Detroit, and create multiple stormwater issues [43]. Natural hazards have been shown to worsen problems of economic inequality [44]. Serious impacts from flooding are the result of a confluence of factors including extreme weather events, robustness of infrastructure and other factors to mitigate flood risk, housing conditions and individual level vulnerability [45,46]. Though significant effort has been directed to major flooding events (e.g., dam breaks, flood wave propagation), little work has been done on recurrent, localized pluvial and non-pluvial flooding in urban contexts.

The City of Detroit, MI, like many post-industrial cities, faces a wide range of converging challenges including population loss, demographic change and decades of financial and political neglect [47]. As of 2020, Detroit’s population is approx. 670,000 people, down from a peak population of 1.6 million people in 1960 [48]. Nearly 80 percent of Detroit residents are African-American. It is among the poorest of large cities in the US with a median household income of $31,000, less than half of the state median income of $72,000 [48]. The City struggles to provide public services to its residents as a result of declining tax revenues, low levels of investment and decline of population density which forces Detroit to provide services using antiquated systems appropriate for a much larger population [49].

A cross-sectional study of 164 homes in northwest Detroit indicated that 64% of homes experienced at least one flooding event in the past year, with many experiencing three or four events [50]. Researchers of a small qualitative study reported on interviews with residents across the city, confirming that flooding was widespread with many potential risk factors, resources for prevention and recovery were uneven, and social, economic, and mental and physical health implications were extensive [51]. The Detroit Office of Sustainability found that residents report that they experience flooding very often (13%), somewhat often (23%), and occasionally (32%) [52]. Though extreme weather events such as that which caused major flooding in Detroit in 2014 have wide ranging acute impacts, recurrent household flooding may be an under-reported phenomenon in a city like Detroit and may be a problem that worsens with climate change [53].

Detroit’s topography is mostly flat, with an overall change of only 33 m between its highest and lowest points. The City’s natural drainage is split between the Detroit and Rouge Rivers, though the natural tributaries were replaced with underground pipes prior to the 1960s. Water runoff and sewage flow through a combined system, discharging more than 58 million liters of treated and untreated sewage [54] which eventually flows into Lake Erie. A result of Detroit’s combined system of rain runoff and sewage discharge is that large rain events can overwhelm the City’s treatment system, causing sewage backflow into homes during storm weather events [55]. Communities along the Detroit River, most notably the Jefferson-Chalmers area, have historically experienced flooding events of various degrees. Aged housing stock, high prevalence of impervious surfaces and high prevalence of basements put Detroit residents at high risk for home flooding [56].

### Research Goals

Recurrent home flooding is an overlooked public health problem that presents a wide range of health risks to populations in economically challenged post-industrial cities like Detroit, Michigan. To inform prevention and recovery efforts, we first describe the extent and frequency of pluvial flooding in Detroit households using data from a house to house survey. As part of this effort, we identify particular locations or areas at high or low risk for household flooding events. Next, we use location information to link households to other data sets to test for associations of household and neighborhood/tract level determinants of household flooding events. Using the results of the first two aims, we create a multivariate model that could be used to predict flooding risk for individual households or households located within specific tracts or areas. Finally, we explore how flooding and factors associated with flooding might determine household asthma prevalence in surveyed households.

We hypothesize that flooding events are concentrated in specific areas and that households that experience flooding events are in proximity to other homes that experience flooding events. We hypothesize that both neighborhood and household level factors will determine pluvial flooding risk. We expect that no one factor in isolation determines household level flooding risk and create a multivariate regression model to predict flood risk using all available variables. Finally, we test the hypothesis that home flooding and factors associated with home flooding also determine household level public health outcomes such as asthma, which may result from exposure to chemicals, bacteria, or mold.

## 2. Materials and Methods

### 2.1. Survey of Home Flooding in Detroit

Wayne State University’s Center for Urban Studies (CUS) works to “improve understanding of and provide innovative responses to urban challenges and opportunities.” As a part of its commitment to serving Detroit and its metropolitan area, the Center maintains a staff of professionals dedicated to identifying opportunities to meet community needs. In 2012, the Center created the Home Safety Assessment (HSA) as a joint effort between AmeriCorps, Wayne State University Center for Urban Studies, FEMA, Detroit Fire Department, Clear Corps, and the Kohl’s Injury Prevention Program through the Children’s Hospital of Michigan. The HSA is a risk assessment survey to determine if a resident’s home has hazardous conditions such as asthma triggers, flood, and moisture/vapor intrusion and the vulnerability of the housing quality. Field staff conduct door-to-door canvasing dividing target zip codes in walk-able target areas. Survey workers approached the home to determine if a household member was present. If no one was home at the time, workers returned at a later date, or scheduled visits on nights and weekends if necessary. To elicit residents’ participation in the project the project offered a small, non-monetary incentives. Survey staff educated residents on ways to increase home safety, and referred residents to partner services. Survey and community outreach activities are ongoing.

This research was deemed exempt from annual review by Institutional Review Boards at the University of Michigan (study number HUM00177793) and Wayne State University (study number 101619B3X).

### 2.2. Neighborhood and Environmental Data

Census tract level measures of poverty and neighborhood age were obtained through the American Community Survey, a sample-based yearly survey of American households maintained by the United States Census [48]. Data for administrative boundaries and locations of waterways were downloaded from the State of Michigan’s GIS Portal [57]. One-meter elevation data was obtained from the Advanced Spaceborne Thermal Emission and Reflection Radiometer (ASTER) Digital Elevation Model from the National Aeronautics and Space Administration (NASA) [58]. Additional residential property information, or parcel data, were extracted from a database of public tax records from real estate analytics company CoreLogic [59]. This database consists of parcel-level property information collected from tax assessor’s records as of 2016 and includes data on the year the structure was built, whether the unit is a rental unit or owner occupied, the assessed value of the home and whether the unit was a multifamily unit.

Latitude and longitude for households were added to the data set using the home address listed in the CUS survey and the geocoder tool inArcMap ver. 10.6.1 [60]. Latitude and longitude coordinates outside of the outer boundary of the the city limits of Detroit were excluded from the analysis. Data from other layers such as elevation and neighborhood information were extracted using the latitude/longitude location of the household obtained from the geocoded address.

#### Statistical Methods

Household representatives were asked if their home had flooded in the past as a result of rainfall. Using this data, we created maps of household locations and flooding events. “Hot” and “Cold” spots for pluvial flooding were determined based on a Getis-Ord statistic at each point. The Getis-Ord Gi* statistic is obtained by examining the value of each location in the context of all surrounding locations. Local sums are compared proportionally to the sum of all values surrounding it and z-scores and p-values are produced [61,62]. Areas of statistically high and low local risk are presented on a map to allow the identification of particular locations of interest. Hot and cold spots are defined as areas where there is statistical evidence that homes that experience/do not experience flooding are in close proximity to other homes that also experience/do not experience flooding. Next, we tested for associations of factors with household flooding status using univariate logistic regression models. Finally, a full, multivariate model of pluvial flooding including all relevant and sufficiently represented predictors was created and a backwards selection was used to construct a multivariate model of best fit.

## 3. Results

### 3.1. Spatial Distribution of Flood Risk in Detroit

**Figure 1:**
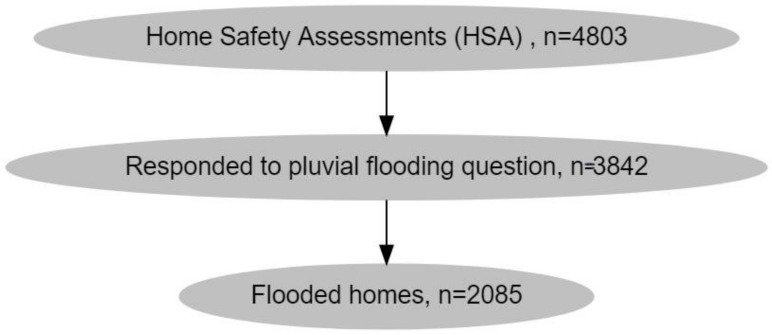
Flowchart of data collection.

**Figure A1:**
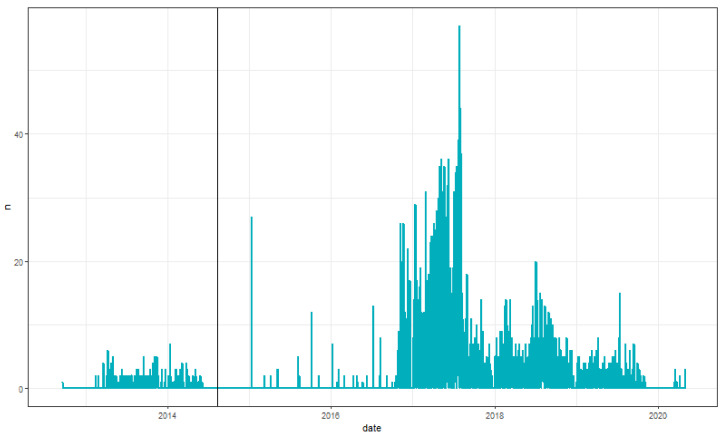
Number of daily home visits over the study period. Vertical line indicates the date of a major flood even in Detroit in 2014.

Survey data from the Center for Urban Studies comprised 4803 households between 18 September 2012 and 5 May 2020. The daily pattern of home visits during this period is shown in Figure A1 in Appendix A. Representatives from 3842 households responded to the question on home flooding due to rainfall. Among these, 2085 (54.26%) reported having experienced flooding. A data collection flow-chart is included in Figure 1.

**Figure 2:**
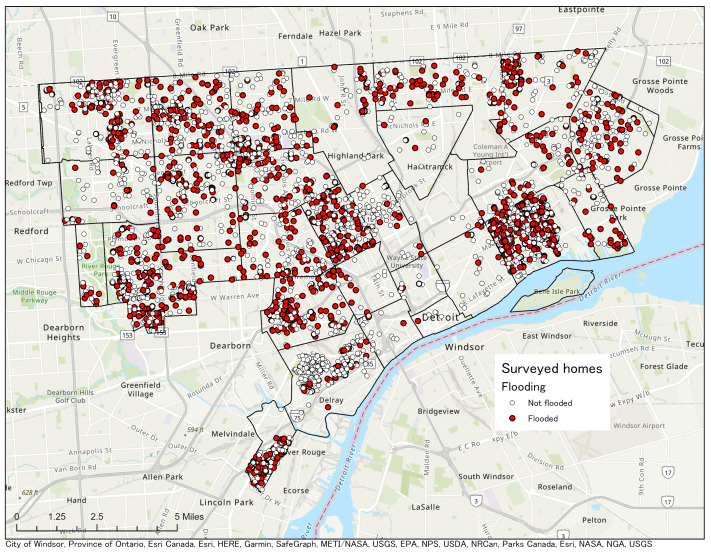
Locations of households which reported that they experienced pluvial flooding. Households that responded that no flooding occurred are not shown.

**Figure 3:**
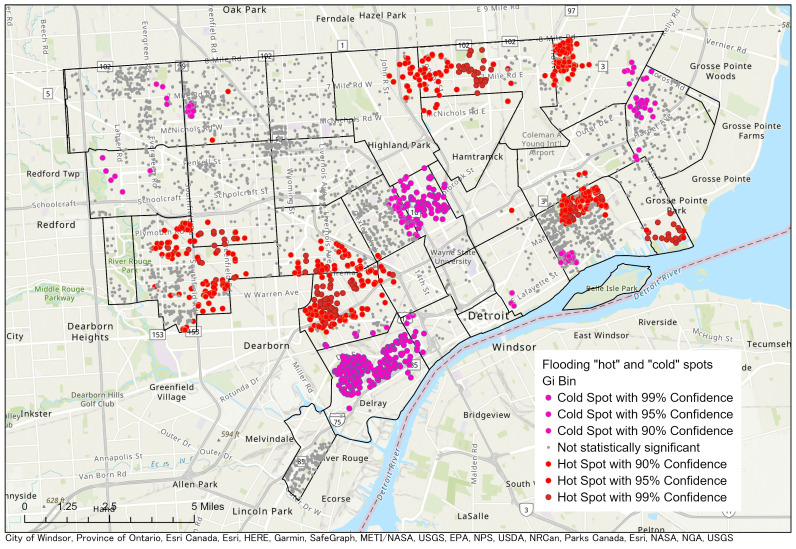
Hot and cold spots of flooding using the Getis-Ord Gi* statistic. Red dots represent “hot” spots, or locations of statistically significant clusters of homes that experienced flooding. Blue does represent clusters of homes that reported not experiencing flooding.

Households that reported flooding were located throughout Detroit. See Figure 2. Though home flooding occurred in all areas of Detroit, “hot spot” analysis using Getis-Ord statistics indicated that there were statistically significant clusters of homes at high and low risk for flooding. There were significant clusters of flooded homes in the southern part of the east side of Detroit, specifically in and around the Jefferson-Chalmers neighborhood. There were also significant clusters of homes that reported never having experienced flooding. See Figure 3.

#### 3.1.1. Household Level Correlates of Home Flooding

In univariate analyses, we found that several factors were significantly associated with flooding risk among surveyed households. Rental homes were more likely to have experienced flooding than owner-occupied homes (OR 1.72 [95% CI 1.49, 1.98]). Unfinished basements were associated with a higher odds of flooding than finished basements (OR 1.25 [95% CI 1.01, 1.54]).

**Figure 4:**
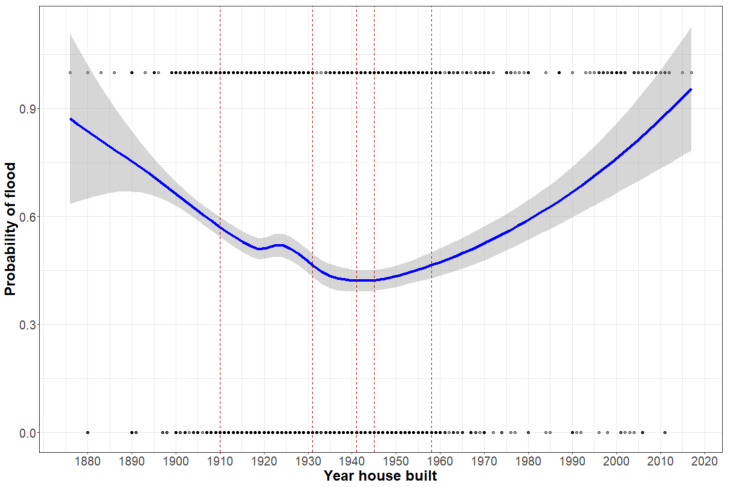
Estimated flooding risk by year of construction using LOESS interpolation method. Vertical lines represent successive building waves (1870–1910, 1911–1930, etc.) used to create housing construction categories.

We also explored whether patterns of flood risk might change with age of home in a non-linear manner. Figure 4 shows a locally estimated scatterplot smoothing (LOESS) based interpolation of flood risk as a function of the year of home construction. Risk for home flooding had a parabolic relationship to home age, with risk being the highest for the oldest homes, decreasing until ~1960 and then rising again. We then categorized the age of the home based on historical waves of home construction [63]. Compared with homes built before 1910, risk for flooding decreases for homes built in waves before 1958. Risk for flooding for home built in the late 20th century and early 21st century is similar to that of the oldest homes in Detroit. See Table 1 for full results.

All of the variables related to housing conditions were predictive of home flooding. Homes with roofs that were reported to be in good condition were found to be less likely to experience flooding (OR 0.59 [95% CI 0.50, 0.70]). Homes with mold on the walls (OR 6.03 [95% CI 5.09, 7.17]), moldy smells (OR 5.51 [95% CI 4.70, 6.49]), basement in disrepair (OR 12.40 [95% CI 10.00, 15.50]), uncapped sewer outlets (OR 2.21 [95% CI 1.91, 2.57]) and occurrence of previous sewer backups (OR 16.10 [95% CI 13.50, 19.30]) were found to be more prone to flooding. See Table 2 for full results.

#### 3.1.2. Neighborhood (Census Tract) and Environmental Correlates of Home Flooding

Of interest to this research was how the surrounding environment raised or lowered the risk of home flooding. The following census tract characteristics were considered and found to not be significantly associated with flood risk: poverty, childhood poverty, percent African-American or Hispanic residents, percent of homes built before 1939, elevation and the distance to the nearest waterway. The percentage of all homes in the census tract that are owner-occupied was inversely ad significantly associated with flood risk (OR 0.92 [95% CI 0.86, 0.98]). See Table 3 for full results.

#### 3.1.3. Multivariate Model of Home Flooding

We tested associations of all variables with home flooding in a multivariate model. Missing data was a problem for many available variables. Any variable with more than 10% of observations missing was excluded from the analysis. Multiple imputation was used to fill in the missing values for the remaining set of predictors [64] so that we could create a “full model” of household and neighborhood factors on home flooding. Using this model, we used backward selection to successively delete variables based on significance until a final, optimal model based on Akaike’s Information Criterion (AIC) [65] was obtained. Variables that were sufficiently represented in the data set to allow model inclusion comprised all census tract level variables such as poverty, racial composition, age of homes in the area, elevation and distance to nearest waterway. Rent/own status and variables on various aspects of housing condition such as leaks and cracks in basement and outer walls were also retained.

After model selection, only a handful of variables were dropped including census tract level poverty and childhood poverty, distance to nearest waterway, elevation and chimney leaks. In the multivariate model we found that rental status (OR 1.18 95% CI [1.09, 1.27]) was associated with flooding even when controlling for other factors. Moldy walls and smells were remained in the model and were significantly associated with flooding. Roofs leaks had the strongest association with flooding (OR 6.84 95% CI [5.33, 8.35]). Sewer backups were left in the model building process as a proxy for the relative quality of the surrounding sewer and flooding infrastructure. Even when controlling for other factors, the odds of flooding given sewer backup were very high (OR 1.67 95% CI [(1.39, 1.94]). The percentage of neighborhood residents who are African American and the age of the neighborhood remained in the model after model selection but this association was not significant. See Table 4 for full results.

#### 3.1.4. Asthma and Home Flooding

A total of 3072 (74.4%) participants reported having at least one adult person in the home who had received a diagnosis of asthma from a health care professional. Having at least one adult with asthma in the home was associated with flooding ((OR 1.42 [95% CI 1.22, 1.64])). Adults with asthma were also very likely to live with a child with asthma (OR 4.54 [95% CI 3.75, 5.50]). Nearly all of the household variables were associated with asthma cases with only the exception of the roof leaks, foundation cracks and window leaks and proper sink and bathtub drainage. None of the census tract level variables were associated with asthma in adults. Other possible predictors of asthma cases such as having a smoker in the home and household breathing problems were also available in the data set and were included in Table 5.

## 4. Discussion

Using a house-to-house survey of homes and residents throughout the city, we have shown that pluvial home flooding is a serious problem that impacts many Detroit residents. We found that while people all over Detroit experience home flooding, certain areas are at particular risk for flooding events. Renters and those living in areas where most homes are not owner occupied live at high risk for flooding. We found that poor housing conditions directly impact risk. When controlling for housing and neighborhood factors, we found that flooding disproportionately impacts communities of color. Finally, we found that flooding is associated with asthma risk in both adults and children.

Our results suggest that home flooding is a far more serious issue than previously thought, disproportionately impacting people who may lack financial means to effectively recover from flooding events and pluvial disasters, and who, due their status as renters, may lack the ability to implement measures to prevent or to mitigate the impacts of floods, such as basement weatherization or roof repairs. This would suggest that a focus on home flooding risk and efforts to improve housing quality should be a priority not only for advocates of urban housing, but also for public health organizations, focusing on programs that educate, work with, and provide resources to renters and incentives to landlords to improve housing quality/maintenance. Research in Germany has also suggested that directly communicating risks of flooding to home owners might encourage them to make improvements to prevent or mitigate flooding risk [66]. Direct communication to landlords, for example, might encourage some to make necessary repairs and improvements to their properties.

We could find no similar studies that use expansive survey efforts to assess regular home flooding in urban areas. We did, however, find that the proportion of homes that experienced flooding in Detroit was in excess of the number of homes estimated to be at risk by other modelling efforts and higher than comparable cities in other Midwestern States [67]. We recommend that surveys of home flooding be done to draw a distinction between flooding as a result of sudden, catastrophic weather events and the regular flooding that residents experience.

Our results indicated that household level factors were far stronger determinants of home flooding than neighborhood factors. Nearly all the household level variables such as basements in need of repair, mold on the walls and moldy smells, cracks in the walls and roof condition were significantly associated with flooding, both in univariate and multivariate analyses. Conversely, neighborhood level measures such as census tract level racial composition, poverty and environmental factors were not found to be associated with flooding. It is possible the low level of racial and socio-economic variation across Detroit prevents the detection of such associations. A study which included the surrounding, more affluent suburbs might have yielded a different set of results. However, our results indicate that within the City of Detroit, policy and intervention efforts which focus on home improvement, particularly for rental properties, might yield positive results.

We found that homes built after 1958 were less likely to have been flooded than home built before 1910, they were more likely to be flooded than homes built between 1910 and 1957. The history of building in Detroit has followed distinct waves of construction, following the city’s economic and social history. Nearly 40% of homes in Detroit were built in two decades prior to the Great Depression and many older homes in Detroit were originally of higher quality than homes which have since been demolished or unoccupied [63]. This may explain the counterintuitive result that newer homes are more likely to be flooded than slightly older homes. Better built homes are usually spaced apart, set back from the street and have more pervious surfaces (yards) to absorb rainfall. Of course, there are exceptions. Homes in the Jefferson-Chalmers neighborhood, which were also built before the Great Depression, comprised a flooding “hot-spot” in our analyses due to its proximity to the river. Newer constructions must meet modern construction standards but comprise denser housing developments in formerly razed areas of the City. More work might be done to determine how housing quality and housing location intersect in the context of flooding risk.

A major limitation of this study was the measure of the flooding outcome. Reporting of flooding occurred only during the in-home visit by the survey team. Thus, flooding events long before the visit that were not in the recollection of the home occupant, and/or flood events after the survey visit were not recorded. Many homes that did not report flooding were visited prior to the city-wide flood of 2014 with the result that impacts from that event were not recorded for those households. Thus, the outcome measurement should be considered an underestimate. Compliance with survey projects is always a challenge. In the case of this study, certain biases should be assumed. For example, given that the present study was described as pertaining to housing conditions and flooding, we might assume that occupants of homes might be more likely to respond who experienced flooding or whose homes were in poor condition.

The flooding data collected also was not specific enough to make recommendations for specific interventions regarding household level flood mitigation. There are multiple causes of in-home flooding, including: sewer back-up, rising water levels of nearby waterways, ponded water around the home, rising groundwater levels around the home and subsequent leakage through foundation and basement, direct inflow through roof leaks, leaks of interior piping, and others. In many cases the flooding may stem from a variety of causes and problems. Future research efforts might ask residents to report incidence of recurrent home flooding to researchers or community leaders and teams could be dispatched to collect detailed information on sources and outcomes.

Detroit experiences the highest health impacts of asthma in Michigan, with the adult asthma rate 29% higher and the rate of asthma-related hospitalizations three times greater than the state average [68]. In-home triggers account for 40% of all asthma episodes [69]. Detroit has been called the “Epicenter of the Asthma Burden,” with at least 11.3% of Detroit children and 15.5% of Detroit adults having diagnosed asthma [70]. We could not find data on household level prevalence of adult asthma but more than three quarters of the homes included in this study had at least one adult with asthma. We found that homes with asthmatic residents were more likely to be flooded. Based on this survey, we cannot conclude that there is a causal link between flooding and asthma. We can, however, conclude that persons with respiratory conditions such as asthma are more likely to live in homes that have been flooded.

Asthma has been associated with flooding in other studies, principally though the development of and exposure to molds and fungus within the home [30,31,32]. Most research on the associations of flooding and health focus on extreme weather events such as Hurricane Katrina in New Orleans, LA [71] and Hurricane Sandy in New Jersey [72]. Regular flooding such as that which occurs in Detroit, however, might be causally associated asthma due to persistent mold problems in the home and constant exposures to molds, fungus and other endotoxins. We note that the high baseline prevalence of asthma in Detroit and the lack of past data on asthma incidence and prevalence complicate efforts to assess causal links between flooding and asthma.

Future studies of this kind could focus on the public health impacts of recurrent flooding in households and how home improvement measures can be implemented as a means of reducing risk for serious health problems. Using a crude measurement of asthma prevalence, our research suggested that housing quality and flooding risk were associated with asthma in both adults and children. Future research should work to better characterize the public health impacts of regular flooding by implementing programs that improve housing conditions, such as basic measures to prevent basement leakage, while looking at basic health indicators. Evidence might suggest that asthma incidence or in asthma related health events decrease after implementing basic improvements such as sealing basements or preventing sewer backflows, which could lead to creative policy that treats home improvements as protecting the public health.

Future survey work should attempt to validate the results of this research. It should also be designed to better understand the causes and outcomes of regular flooding in Detroit. These efforts should be conducted on a regular basis, but should also mobilize during or after times of intense precipitation or known flooding events. Any survey work should also collect quantitative data such as high water mark and source location, and also collect detailed information on individual, home and neighborhood conditions that might contribute to flooding. Future studies might assess these factors over time and test for association with climate and rainfall patterns for forecasting and prediction. Qualitative information on residents’ experiences and ideas could help inform community and government efforts to mitigate flood and support flood victims. The often overlooked public health implications of regular flooding should, however, be a major focus of future research work to ensure equitable prevention and response.

## 5. Conclusions

Pluvial household flooding is a serious problem for Detroit residents though risk is clustered in certain areas of the city. People living in rental units experience higher risks for household flooding than those in owner occupied units and poor housing conditions are predictive of water entering the home. Household level factors and housing quality were found to be far more predictive of home flooding than neighborhood factors, suggesting that interventions which focus on home improvement might be effective in mitigating flooding risk. Finally, we found that flooding is associated with public health outcomes such as asthma. These results suggest that flooding in Detroit is an important environmental justice and public health issue worthy of further attention to researchers and public policy advocates.

## Figures and Tables

**Table 1:** Comparison of household factors by flooding status. Counts and percentages of categorical predictors are noted. Continuous variables are shown as means with standard deviation in parentheses. Comparisons of flooding with categorical predictors are made using Chi-square tests. Comparisons of flooding for continuous predictors are made using t-tests of means. Odds ratios for continuous predictors are based on standardized values (z-scores). presented for categorical predictors. Both continuous and categorical forms of the year the house was built are presented.

	[ALL]	No Flood	Flood	OR	p.ratio
	N = 3842	N = 1757	N = 2085		
Own or rent home:					
Own	2458 (64.0%)	1241 (70.6%)	1217 (58.4%)	Ref.	Ref.
Rent	1225 (31.9%)	456 (26.0%)	769 (36.9%)	1.72 [1.49;1.98]	<0.001
Approximate year house was built	1929 (22.7)	1931 (20.0)	1927 (24.8)	0.99 [0.99;1.00]	<0.001
Year house built (categories):					
Pre 1910	653 (17.0%)	219 (12.5%)	434 (20.8%)	Ref.	Ref.
1910–1930	986 (25.7%)	496 (28.2%)	490 (23.5%)	0.50 [0.41;0.61]	<0.001
1931–1940	319 (8.30%)	192 (10.9%)	127 (6.09%)	0.33 [0.25;0.44]	<0.001
1941–1945	88 (2.29%)	47 (2.68%)	41 (1.97%)	0.44 [0.28;0.69]	<0.001
1946–1957	540 (14.1%)	297 (16.9%)	243 (11.7%)	0.41 [0.33;0.52]	<0.001
1958 or later	197 (5.13%)	83 (4.72%)	114 (5.47%)	0.69 [0.50;0.96]	0.029
Building sq ft	1367 (720)	1354 (690)	1379 (746)	1.04 [0.97;1.11]	0.325
Living space sq ft	1364 (712)	1353 (689)	1374 (732)	1.03 [0.96;1.11]	0.404
Basement sq ft	819 (274)	807 (261)	830 (285)	1.09 [1.01;1.17]	0.019
Type of basement:					
Finished	402 (12.5%)	211 (13.8%)	191 (11.3%)	Ref.	Ref.
Unfinished	2819 (87.5%)	1322 (86.2%)	1497 (88.7%)	1.25 [1.01;1.54]	0.036

**Table 2:** Housing conditions and flooding. Housing condition measurements were self reported by residents and confirmed by survey workers where possible.

	[ALL]	No Flood	Flood	OR	p.ratio
	**N = 3842**	**N = 1757**	**N = 2085**		
Roof in good condition:	680 (17.7%)	378 (21.5%)	302 (14.5%)	0.59 [0.50;0.70]	<0.001
Mold on walls of basement:	2720 (70.8%)	973 (55.4%)	1747 (83.8%)	6.03 [5.09;7.17]	0.000
Moldy smells:	2633 (68.5%)	925 (52.6%)	1708 (81.9%)	5.51 [4.70;6.49]	0.000
Basement needs repair:	2639 (68.7%)	948 (54.0%)	1691 (81.1%)	12.4 [10.0;15.5]	0.000
Roof leaks:	2997 (78.0%)	1304 (74.2%)	1693 (81.2%)	2.04 [1.67;2.49]	<0.001
Window leaks:	3248 (84.5%)	1465 (83.4%)	1783 (85.5%)	1.64 [1.24;2.18]	<0.001
Chimney leaks:	3383 (88.1%)	1538 (87.5%)	1845 (88.5%)	2.06 [1.31;3.32]	0.002
Plumbing leaks:	3131 (81.5%)	1360 (77.4%)	1771 (84.9%)	2.88 [2.26;3.69]	0.000
No leaks:	1068 (27.8%)	647 (36.8%)	421 (20.2%)	0.42 [0.36;0.49]	0.000
Foundation cracks:	2945 (76.7%)	1243 (70.7%)	1702 (81.6%)	2.74 [2.26;3.35]	0.000
Sinks and bathtub drain properly:	1026 (26.7%)	541 (30.8%)	485 (23.3%)	0.67 [0.58;0.78]	<0.001
Uncapped sewer outlets:	2542 (66.2%)	1034 (58.9%)	1508 (72.3%)	2.21 [1.91;2.57]	0.000
Ever had sewer backups:	2465 (64.2%)	638 (36.3%)	1827 (87.6%)	16.1 [13.5;19.3]	0.000

**Table 3:** Neighborhood and environmental determinants of recurrent home flooding. Odds ratio for continuous measures produced using standardized values (z-scores).

	[ALL]	No Flood	Flood	OR	p.ratio
	**N = 3842**	**N = 1757**	**N = 2085**		
Census tract poverty (%)	43.1 (11.5)	43.0 (11.4)	43.1 (11.5)	1.02 [0.95;1.08]	0.648
Census tract under 18 poverty (%)	59.6 (17.5)	59.4 (17.6)	59.8 (17.4)	1.02 [0.96;1.09]	0.458
Percent African-American	86.0 (24.1)	85.8 (23.7)	86.2 (24.4)	1.02 [0.94;1.10]	0.671
Percent Hispanic	7.52 (18.8)	7.28 (18.4)	7.73 (19.2)	1.03 [0.95;1.12]	0.499
Distance to nearest waterway (m)	4.85 (2.99)	4.92 (3.04)	4.79 (2.95)	0.96 [0.90;1.02]	0.186
Elevation (m)	189 (7.22)	189 (7.12)	189 (7.30)	0.97 [0.91;1.03]	0.286
Percent of homes owner occupied	51.0 (13.8)	51.7 (13.0)	50.5 (14.3)	0.92 [0.86;0.98]	0.008
Percent of home built before 1939	39.5 (24.3)	38.9 (24.1)	40.0 (24.5)	1.05 [0.98;1.11]	0.166

**Table 4:** Full and reduced models of flooding using all sufficiently represented variables. Reduced model found through backwards selection through AIC. All continuous variables have been standardized and thus odds ratios for these variables represent changes in odds given unit changes in z-scores.

	*Dependent Variable:*	
	Flooding	
	Full Model	Reduced Model
	(1)	(2)
Census tract poverty (%)	1.029 (0.895, 1.163)	
Census tract under 18 poverty (%)	0.968 (0.846, 1.090)	
Percent African-American	1.210 ${}^{*}$ (0.967, 1.453)	1.073 (0.977, 1.168)
Distance to nearest waterway (m)	1.138 (0.904, 1.371)	
Elevation (m)	0.975 (0.870, 1.081)	
Homes owner occupied (%)	1.009 (0.891, 1.128)	
Home built before 1939 (%)	0.941 (0.865, 1.017)	0.941 (0.869, 1.014)
Own or rent home	1.164 ${}^{***}$ (1.059, 1.270)	1.179 ${}^{***}$ (1.087, 1.270)
Mold on walls of basement	1.547 ${}^{***}$ (1.296, 1.797)	1.551 ${}^{***}$ (1.302, 1.801)
Moldy smells	2.017 ${}^{***}$ (1.539, 2.495)	2.037 ${}^{***}$ (1.556, 2.518)
Basement needs repair	1.783 ${}^{***}$ (1.380, 2.187)	1.777 ${}^{***}$ (1.376, 2.179)
Roof leaks	6.753 ${}^{***}$ (5.250, 8.256)	6.836 ${}^{***}$ (5.323, 8.349)
Window leaks	0.965 (0.710, 1.221)	
Chimney leaks	0.687 ${}^{**}$ (0.430, 0.945)	0.684 ${}^{**}$ (0.446, 0.922)
Plumbing leaks	0.809 (0.335, 1.283)	
Foundation cracks	1.225 (0.854, 1.597)	
Sinks and bathtub drain properly	1.339 ${}^{**}$ (0.998, 1.680)	1.380 ${}^{***}$ (1.045, 1.716)
Uncapped sewer outlets	0.916 (0.758, 1.073)	
Ever had sewer backups	1.663 ${}^{***}$ (1.384, 1.942)	1.668 ${}^{***}$ (1.390, 1.946)
Gaps large enough for animals and insects	1.282 ${}^{**}$ (1.029, 1.535)	1.300 ${}^{***}$ (1.047, 1.553)
Constant	0.059 ${}^{***}$ (0.024, 0.095)	0.052 ${}^{***}$ (0.032, 0.072)
Observations	3842	3842
Log Likelihood	−2088.269	−2090.988
Akaike Inf. Crit.	4218.538	4205.976

Note: * *p* < 0.1; ** *p* < 0.05; *** *p* < 0.01.

**Table 5:** Prevalence and determinants of asthma in at least one adult member of the household. Odds ratios for continuous measures from standardized values (z-scores).

	No	Yes	OR	*p*
	N = 1057	N = 3072		
Experienced flooding	448 (47.5%)	1542 (56.2%)	1.42 [1.22;1.64]	<0.001
Child in the home with asthma: Yes	497 (62.2%)	2005 (88.2%)	4.54 [3.75;5.50]	0.000
Breathing problems: Yes	389 (39.7%)	2355 (80.8%)	6.40 [5.47;7.50]	0.000
Smoker in the home: Yes	514 (49.9%)	1774 (59.5%)	1.47 [1.28;1.70]	<0.001
Year house was built:				
Pre 1910	137 (18.3%)	512 (22.9%)	Ref.	Ref.
1910–1930	275 (36.7%)	780 (34.9%)	0.76 [0.60;0.96]	0.020
1931–1940	93 (12.4%)	251 (11.2%)	0.72 [0.53;0.98]	0.037
1941–1945	28 (3.74%)	84 (3.76%)	0.80 [0.51;1.30]	0.358
1946–1957	170 (22.7%)	444 (19.9%)	0.70 [0.54;0.91]	0.007
1958 or later	46 (6.14%)	165 (7.38%)	0.96 [0.66;1.41]	0.825
Roof in good condition	186 (23.9%)	490 (21.8%)	0.88 [0.73;1.07]	0.214
Mold on walls of basement	637 (69.4%)	2020 (76.3%)	1.42 [1.20;1.68]	<0.001
Moldy smells: Yes	610 (66.5%)	1951 (73.5%)	1.40 [1.19;1.64]	<0.001
Basement needs repair	619 (70.3%)	1937 (77.2%)	1.43 [1.21;1.70]	<0.001
Roof leaks	830 (85.6%)	2402 (86.3%)	1.06 [0.86;1.31]	0.579
Window leaks	896 (92.4%)	2617 (94.0%)	1.30 [0.97;1.71]	0.079
Chimney leaks	937 (96.6%)	2727 (98.0%)	1.69 [1.08;2.59]	0.022
Plumbing leaks	854 (88.0%)	2521 (90.6%)	1.30 [1.03;1.64]	0.028
No leaks	338 (34.8%)	850 (30.5%)	0.82 [0.70;0.96]	0.013
Foundation cracks	758 (83.8%)	2221 (85.7%)	1.16 [0.94;1.42]	0.169
Sinks and bathtub drain properly	297 (28.6%)	772 (25.8%)	0.87 [0.74;1.02]	0.077
Uncapped sewer outlets	624 (68.1%)	1908 (72.0%)	1.20 [1.02;1.41]	0.028
Ever had sewer backups	581 (62.8%)	1796 (66.9%)	1.20 [1.02;1.40]	0.025
Gaps large enough for animals and insects	672 (71.6%)	2089 (77.4%)	1.36 [1.15;1.60]	<0.001
Census tract poverty (%)	42.7 (11.4)	42.9 (11.7)	1.00 [1.00;1.01]	0.639
Census tract under 18 poverty (%)	59.1 (17.8)	59.2 (17.8)	1.00 [1.00;1.00]	0.888
Percent African-American (%)	85.7 (24.3)	85.5 (24.5)	1.00 [1.00;1.00]	0.838
Distance to nearest waterway (m)	4.93 (3.09)	4.85 (3.01)	0.99 [0.97;1.01]	0.479
Elevation (m)	189 (7.22)	189 (7.18)	1.00 [0.99;1.01]	0.488
Homes owner occupied (%)	51.3 (13.3)	50.8 (14.1)	1.00 [0.99;1.00]	0.296
Homes built before 1939 (%)	39.1 (24.1)	39.0 (24.2)	1.00 [1.00;1.00]	0.958

## Data Availability

Supporting data is available upon reasonable request. The privacy of participants will be respected.
